# Beneficial Effects of Sarpogrelate and Rosuvastatin in High Fat Diet/Streptozotocin-Induced Nephropathy in Mice

**DOI:** 10.1371/journal.pone.0153965

**Published:** 2016-04-20

**Authors:** Dong-hyun Kim, Bo-hyun Choi, Sae-Kwang Ku, Jeong-hyeon Park, Euichaul Oh, Mi-Kyoung Kwak

**Affiliations:** 1 College of pharmacy, The Catholic University of Korea, Bucheon, Gyeonggi-do, 420-743, Republic of Korea; 2 College of Korean Medicine, Daegu Haany University, Gyeongsan, Gyeonsangbuk-do, 712-715, Republic of Korea; University of Florida, UNITED STATES

## Abstract

Chronic kidney disease (CKD) is a major complication of metabolic disorders such as diabetes mellitus, obesity, and hypertension. Comorbidity of these diseases is the factor exacerbating CKD progression. Statins are commonly used in patients with metabolic disorders to decrease the risk of cardiovascular complications. Sarpogrelate, a selective antagonist of 5-hydroxytryptamine (5-HT) 2A receptor, inhibits platelet aggregation and is used to improve peripheral circulation in diabetic patients. Here, we investigated the effects of sarpogrelate and rosuvastatin on CKD in mice that were subjected to a high fat diet (HFD) for 22 weeks and a single low dose of streptozotocin (STZ, 40 mg/kg). When mice were administrated sarpogrelate (50 mg/kg, *p*.*o*.) for 13 weeks, albuminuria and urinary cystatin C excretion were normalized and histopathological changes such as glomerular mesangial expansion, tubular damage, and accumulations in lipid droplets and collagen were significantly improved. Sarpogrelate treatment repressed the HFD/STZ-induced CD31 and vascular endothelial growth factor receptor-2 expressions, indicating the attenuation of glomerular endothelial proliferation. Additionally, sarpogrelate inhibited interstitial fibrosis by suppressing the increases in transforming growth factor-β1 (TGF-β1) and plasminogen activator inhibitor-1 (PAI-1). All of these functional and histological improvements were also seen in rosuvastatin (20 mg/kg) group and, notably, the combinatorial treatment with sarpogrelate and rosuvastatin showed additive beneficial effects on histopathological changes by HFD/STZ. Moreover, sarpogrelate reduced circulating levels of PAI-1 that were elevated in the HFD/STZ group. As supportive *in vitro* evidence, sarpogrelate incubation blocked TGF-β1/5-HT-inducible PAI-1 expression in murine glomerular mesangial cells. Taken together, sarpogrelate and rosuvastatin may be advantageous to control the progression of CKD in patients with comorbid metabolic disorders, and particularly, the use of sarpogrelate as adjunctive therapy with statins may provide additional benefits on CKD.

## Introduction

Chronic kidney disease (CKD) is defined by the gradual reduction in glomerular filtration rate (GFR) and elevation in urinary albumin excretion [1, 2]. CKD is a progressive disease that proceeds to end-stage renal disease (ESRD) in which patients are in need of renal dialysis or kidney transplantation [3]. It has been estimated that more than 10% of adults have CKD and the prevalence is much greater in high-risk populations such as elderly people [1, 4]. The significance of CKD can be found in its strong correlation with the cardiovascular diseases: CKD patients exhibited higher rates of incidence in myocardial infarction, heart failure, and stroke [5, 6].

As causative diseases of CKD, metabolic disorders such as obesity, diabetes mellitus, hyperlipidemia, and hypertension showed strong associations with CKD [1, 7]. Diabetic CKD is the leading cause of ESRD, and CKD prevalence in diabetic patients was known to be up to 40% [8]. In a large cohort study of health men, increased body mass index showed a significantly higher CKD risk after a 14-year follow up [9]. The decline of renal function in middle-aged men with dyslipidemia was accelerated by the increased levels of the low-density lipoprotein (LDL)-high-density lipoprotein (HDL) ration [10]. It is noteworthy that metabolic disorders are usually comorbid diseases. In the cohort study of the National Health and Nutrition Examination Survey (NHANES), 61% of patients with type 2 diabetes were obese and 46% of diabetic patients had lipid abnormalities [11]. Comorbid illness affected CKD prevalence: patients with comorbidities of metabolic disorders have a greater risk for CKD aggravation than patients with s single disease do [12]. In a cohort of patients with CKD stage 3, multimorbidity of metabolic disorders showed the association with the GFR decrease [13]. As an animal model of metabolic disorders, high-fat diet (HFD) has been used widely [14]. Particularly, it was shown that HFD (16 weeks) in C57BL/6JJ mice developed a prediabetic nephropathy that accompanied albuminuria, glomerular enlargement, and marginal levels of collagen deposition [15]. In addition, HFD-induced nephropathy has been exacerbated by the addition of low dose of streptozotocin (STZ), indicating that the coexistence of metabolic diseases can accelerate CKD [16, 17].

Tubulointerstitial fibrosis is one of major features and a reliable predictor of progressive diabetic CKD [18]. In fibrotic CKD lesions, excessive accumulation of extracellular matrix (ECM) such as collagen, fibronectin and laminin is observed [19, 20]. It has been demonstrated that transforming growth factor-β1 (TGF-β1) is an important mediator that produces ECM in various renal cell types such as fibroblasts, tubular epithelial cells, and mesangial cells [21, 22]. Plasminogen activator inhibitor-1 (PAI-1) is a well-known downstream target of TGF-β1 signaling [23, 24]. PAI-1 prevents the degradation of ECM proteins by inhibiting the conversion of tissue plasminogen to plasmin, a matrix metalloproteinase (MMP)-like protease [23]. In addition, the level of plasma PAI-1 correlated with the pathologies of diabetes, obesity, and cardiovascular disease [25–28].

Platelet serotonin (5-hydroxytryptamine; 5-HT) is released to the systemic circulation where it mediates multiple deleterious functions, including vascular smooth muscle constriction and proliferation, and platelet aggregation through the 5-HT_2A_ receptor [29, 30]. In diabetic patients, platelets are activated and therefore the plasma 5-HT level is high and platelet aggregation in enhanced [31, 32]. Sarpogrelate, a selective 5-HT_2A_ receptor antagonist, is an anti-platelet drug and is currently used in patients with peripheral arterial disease and diabetes [33]. Based on the association of 5-HT with diabetic vasculature, a beneficial effect of sarpogrelate on metabolic disorder-induced CKD can be hypothesized. The statin therapy is commonly used for the patients with metabolic disorders to reduce the risk of cardiovascular events [34]. Based on the clinical observations showing the linkage between dyslipidemia and CKD, the potential benefits of statin therapy has gained great attentions [35, 36]. Based on these, in order to investigate the potential therapeutic effects of sarpogrelate and rosuvastatin on CKD, we used the HFD-fed C57BL/6JJ mice that were administered with a single and low dose of STZ as a model of metabolic disorder-induced CKD. In addition, because many patients with metabolic disorders are under the statin treatment to treat comorbid dyslipidemia and to prevent cardiovascular events, we also asked the potential renobeneficial effects of combination of sarpogrelate and rosuvastatin in these mice.

## Materials and Methods

### Materials

Sarpogrelate hydrochloride was obtained from Chemtros (Ansan, Republic of Korea), and rosuvastatin calcium was from Yuhan Chemical Inc. (Ansan, Republic of Korea). Antibodies for vascular endothelial growth factor receptor-2 (VEGFR-2), PAI-1, TGF-β1, and collagen IV were purchased from Abcam (Cambridge, MA, UK). Antibodies that recognize glyceraldehyde 3-phosphate dehydrogenase (Gapdh), β-tubulin, and secondary antibodies (goat anti-rabbit and goat anti-mouse) were obtained from Santa Cruz Biotechnology, Inc. (Dallas, TX, USA). Caspase-3 and CD31 antibodies were from BD Sciences (San Jose, CA, USA) and Cell Signaling Technology, Inc. (Danvers, MA, USA), respectively. Most reagents such as STZ and 5-HT were purchased from Sigma-Aldrich Co. LLC (Saint Louis, MO, USA).

### Animal experiment

C56BL/6J male mice at 7-weeks of age were purchased from Orient Bio Inc. (Gyeonggi-do, Republic of Korea). After a week of quarantine, mice were maintained in a specific pathogen free environment with a 12-h light/dark cycle. The animal experimental protocol was approved by the Institutional Ethics Committee on Animal Care and Experimentation at the Catholic University of Korea (Approval number: 2014–021). A total of 34 mice was randomly divided into five groups: the normal fat diet (NFD) group (n = 4), the HFD/STZ control group (n = 5), the HFD/STZ + sarpogrelate group (n = 8), the HFD/STZ + rosuvastatin (n = 7), and the HFD/STZ + sarpogrelate + rosuvastatin group (n = 7). The NFD and HFD groups were continuously fed either a normal chow diet (fat 18%, protein 24%, and carbohydrates 58%; Harlan Laboratories, Indianapolis, IN, USA) or a HFD with a composition of 60% fat, 20% protein, and 20% carbohydrate (Research Diets, New Brunswick, NJ, USA) for 22 weeks. After 7 weeks of a HFD, the HFD/STZ groups received STZ (40 mg/kg) dissolved in sodium citrate (pH 4.5) via intraperitoneal injection. The NFD group was injected with the sodium citrate vehicle. At Week 9 of the HFD, mice were administered either vehicle (0.9% saline), sarpogrelate (50 mg/kg dissolved in saline), and/or rosuvastatin (20 mg/kg dissolved in saline) via oral gavage for 13 weeks. Sarpogrelate was administered 6 days a week and rosuvastatin was treated 5 days a week. After the completion of the HFD for 22 weeks, mice were anesthetized with diethyl ether and serum samples were performed by cardiac puncture. Kidneys were harvested following a phosphate buffered saline (PBS) perfusion and then formalin-fixed or frozen for subsequent analyses.

### Measurements of serum glucose and lipids

At 5 days after STZ injection and during week 15 of the HFD, mice were fasted overnight (12 h) and blood samples were collected by cutting the tip of the tail. Fasting serum glucose levels were measured using an Accu-Chek blood glucose meter (Roche Diagnostics, Castle Hill, Australia). The levels of serum cholesterol, triglycerides, and blood urea nitrogen (BUN) were determined in serum with an Idexx VetTest Biochemical Analyzer (Westbrook, ME, USA). The serum PAI-1 levels, which include active, inactive, and latent forms of PAI-1, were assessed using a PAI-1 Total Mouse ELISA kit (Abcam) according to the manufacturer’s instructions.

### Measurements of urine albumin and cystatin C

Mice were placed in metabolic cages at week 20 of the HFD and were adapted for 24 h. Two to three mice were housed per metabolic cage and pooled urine samples were collected for 15 h. For each group, three different collections were performed. The urinary albumin level was determined with an Albuwell M ELISA kit (Exocell Inc., PA, USA) according to the manufacturer’s instructions. The urinary cystatin C level was quantified using the Mouse/Rat Cystatin C Quantikine kit (R&D Systems, MN, USA). The measured albumin and cystatin C levels were normalized corresponding urine creatinine levels, which were determined using the Idexx VetTest Biochemical Analyzer.

### Determination of renal histopathology

Kidneys were fixed in 10% neutral buffered formalin, embedded in paraffin, serially sectioned (3~4 μm), and stained with hematoxylin and eosin (H&E) for general observations of histopathology with a light microscope (Nikkon, Tokyo, Japan) [37, 38]. Histological evaluation was performed in the central zone of the cortex. The numbers of glomeruli with mesangial expansion and vasodilation, and the numbers of vacuolated renal tubules were counted using a computer-based automated image analyzer (iSolution FL ver 9.1, IMT i-solution Inc., Vancouver, Quebec, Canada) according to previously established methods [37]. These histomorphological measurements were represented as the percentage number of glomeruli or tubules. Two histological fields for each kidney tissue were analyzed. The histopathologist was blind to treatment groups when analysis was made.

### Oil red O and collagen staining

Portions of kidney were dehydrated in a 30% sucrose solution and then sectioned with a cryostat for Oil red O staining. For the staining of collagen fibers, paraffin-embedded tissue slices were stained with Sirius red. Collagen occupied regions indicated by Sirius red staining (% of stained area) were quantified as established previously [37]. Areas of renal tubules with lipid droplet deposition were analyzed in Oil red O stained. Two histological fields were analyzed in each kidney tissue.

### Immunohistochemical analysis

The immunoreactivities against the apoptotic marker caspase-3, endothelial marker CD31 and Vegfr, and pro-fibrotic markers TGF-β1 and PAI-1 were quantified in the kidney cortex using the avidin-biotin-peroxidase complex (ABC) and peroxidase substrate kit (Vector Labs, Burlingame, CA, USA). Briefly, tissue slices were heated (95–100°C) in 10 mM citrate buffer (pH 6.0) for epitopes retrieval. Endogenous tissue peroxidase activity was blocked by incubating in methanol containing 0.3% H_2_O_2_ for 30 min. The normal horse serum blocking solution was incubated for 1 h in a humidified chamber to block non-specific binding of immunoglobulin. Endogenous avidin and biotin were blocked by avidin/biotin blocking reagent (Dako, Carpinteria, CA, USA). The primary antibody incubation was performed overnight at 4°C in a humidified chamber, followed by incubation at room temperature with the appropriate biotinylated secondary antibody for 1 h and the ABC reagent. Finally, tissue sections were incubated with peroxidase substrate for 3 min and immunoreactive areas were quantified with an automated image analyzer [39].

### Western blot analysis

The other halves of frozen kidney sections were homogenized in radioimmunoprecipitation assay (RIPA) buffer containing a protease inhibitor cocktail (Sigma-Aldrich Co. LLC) with a tissue homogenizer (IKA-Werke GmbH & Co. KG, Staufen, Germany). The protein homogenates were centrifuged twice at 10,000 *g* for 20 min at 4°C. The supernatants were obtained and the protein concentrations were assessed with Bicinchoninic Acid Kits (Thermo Fisher Scientific Inc. Waltham, MCA, USA). Proteins were separated on 10–12% sodium dodecyl sulfate-polyacrylamide gels and transferred to nitrocellulose membranes (GE Healthcare Biosciences, Pittsburgh, PA, USA). Membranes were blocked by incubation in 1% skim milk or 3% bovine serum albumin for 1 h. Incubation with the appropriate primary antibody performed overnight, followed by incubation with the appropriate secondary antibody for 1 h. Chemiluminescent images were captured with an ImageQuant LAS 4000 Mini (GE Healthcare Biosciences).

### Cell Culture

The mouse glomerular mesangial cell line SV40 MES 13 (MES13) was obtained from the American Type Culture Collection (Manassas, VA, USA). MES13 was maintained in a medium containing a 3:1 mixture (v/v) of Dulbecco's modified Eagle medium and Ham’s F-12 medium (GE HealthCare Life Sciences, Logan, UT, USA) with 5% fetal bovine serum (GE HealthCare Life Sciences) supplementation. The cells were grown at 37°C in a humidified 5% CO_2_ atmosphere.

### MTT assay

Cells were grown in 96-well plates at a density of 4 × 10^5^ cells per well. After drug treatment, the cells were incubated with an 3-(4,5-dimethylthiazol-2-yl)-2,5-diphenyltetrazoliumbromide (MTT) solution (2 mg/mL) for 4 h. The reduced formazan was solubilized in dimethyl sulfoxide (DMSO) and optical absorbance was determined with a SPECTROstar Nano microplate reader (BMG Labtech, Ortenberg, Germany) at 540 nm.

### RNA isolation and real-time reverse transcriptase (RT)-polymerase chain reaction (PCR) analysis

Total RNA was isolated from the cells with TRIzol reagent (Thermo Fisher Scientific Inc.). cDNA synthesis was performed by an RT reaction as described previously [40]. For relative quantification of the mRNA levels, PCR analyses were performed with a Roche LightCycler (Roche Diagnostics Deutschland GmbH). The PCR primer sequences used for murine *pai-1* were 5’-ACGTTGTGGAACTGCCCTAC-3’ AMF 5’-GCCAGGGTTGCACTAAACAT-3’ (Bioneer Corporation, Daejeon, Republic of Korea).

### Statistical Analysis

Multiple comparison tests for different treatment groups were conducted. Variance homogeneity was examined using the Levene test. If the Levene test indicated no significant deviations from variance homogeneity, the data were analyzed by a one-way analyses of variance (ANOVA) followed by the least-significant differences (LSD) multi-comparison test to determine which pairs of groups were significantly different (analysis of vacuolated renal tubules, immunohistochemcial analysis of PAI-1). In the case of significant deviations from variance homogeneity in Levene’s test, the Kruskal-Wallis H test was conducted and then, followed by the Mann-Whitney U (MW) test. Statistical analyses were conducted using SPSS for Windows (Release 14.0K, SPSS Inc., Chicago, IL, USA). Differences were considered significant at *P* < 0.05.

## Results

### Establishment of HFD/STZ-induced CKD mouse model

**Fig 1:**
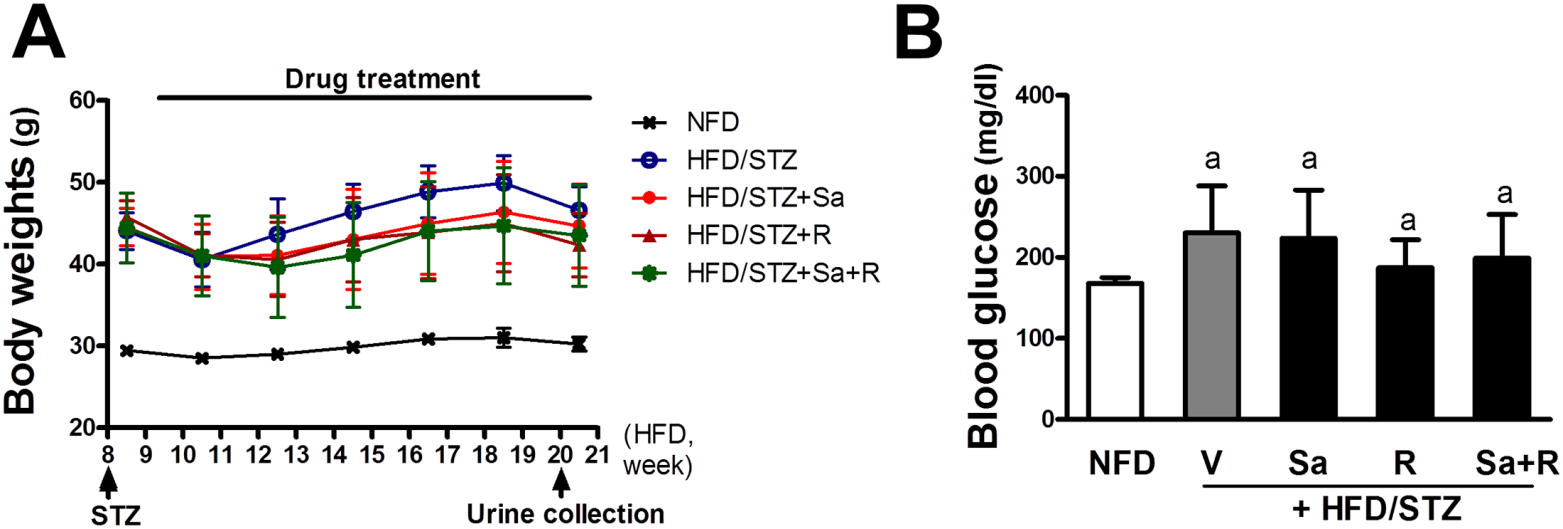
Establishment of the HFD/STZ mouse model. (A) After STZ treatment at Week 8, body weight changes in HFD-fed mice were monitored once per week. HFD was stared a Week 1 and was continued for 22 weeks. Drug treatments began at Week 9 and continued for 13 weeks. (B) Fasting blood glucose levels were monitored at Week 15 of HFD. Mice were fasted overnight (12 h) and blood glucose levels were measured with an Accu-Chek blood glucose meter. The data are means ± standard deviation (SD) from 4–8 samples. ^a^P < 0.05 compared with the NFD group.

In order to establish a CKD model, C57BL/6JJ mice were subjected to a NFD or a 60%-fat diet at 8 weeks of age. During week 8 of the HFD, the mean body weights of mice were 29 g and 44 g in NFD and HFD groups, respectively. At Week 8 of HFD, a single and low dose of STZ (40 mg/kg, *p*.*o*.) was injected into HFD group mice to accelerate CKD progression. It was shown that a single administration of low dose STZ (100 mg/kg) developed non-insulin dependent diabetes in mice [41]. At 5 days after STZ treatment, the level of fasting blood glucose was elevated from 166 mg/dl (NFD) to 220 mg/dl (HFD/STZ), whereas the level of the HFD only group was 208 mg/dl (data not shown). One week after STZ injection, treatment with sarpogrelate and rosuvastatin were started and proceeded to week 22, along with the HFD. The body weights of STZ-treated mice decreased slightly week 10. However, all groups showed steady increases in body weight after week 11, and there were no noticeable differences between treatment groups (Fig 1A). During the treatment period, the fasting blood glucose level was determined at week 15. The results show that the HFD-STZ group displayed a higher level of blood glucose than the NFD mice (Fig 1B). The HFD only group showed a lower fasting glucose level than HFD/STZ group although no statistical significance was observed (unpublished data), which implied that STZ treatment contributed to CKD acceleration. Drug treatment did not show statistically significant changes in HFD/STZ-induced glucose increase.

### Sarpogrelate and rosuvastatin attenuate renal dysfunction in HFD/STZ mice

**Fig 2:**
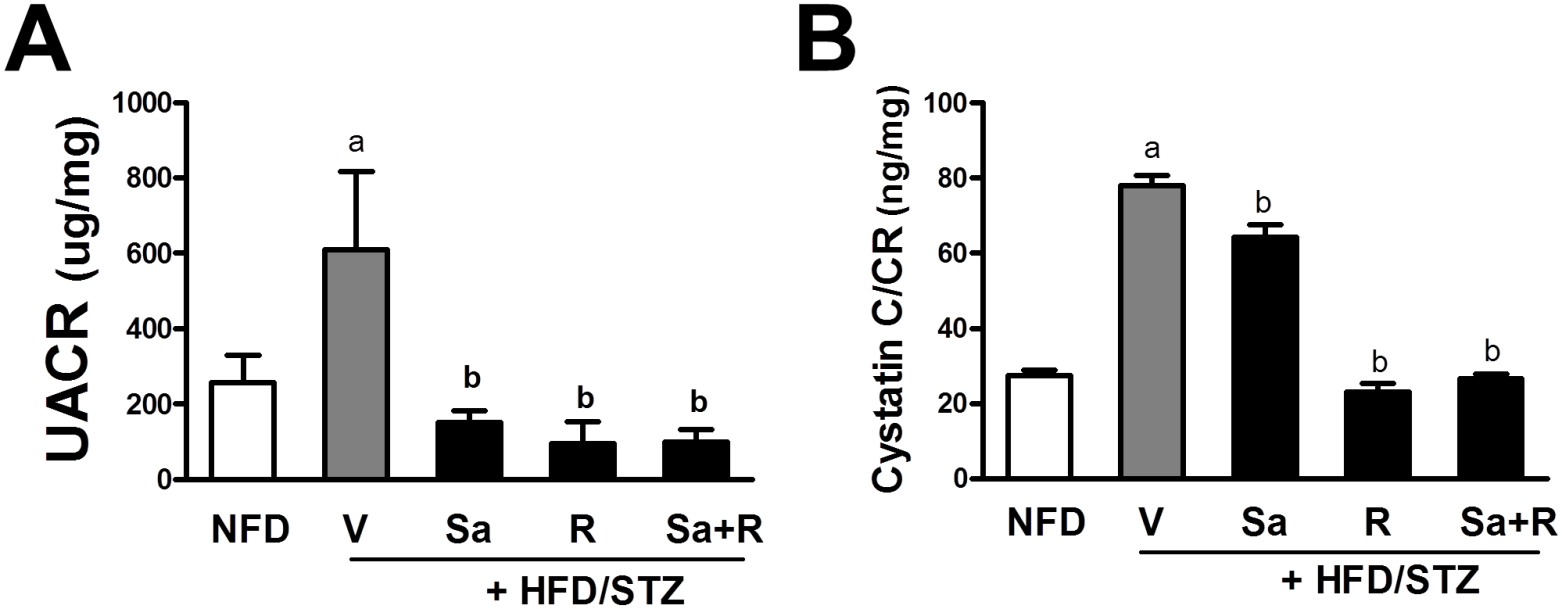
Effects of sarpogrelate (Sa) and rosuvastatin (R) on HFD/STZ-induced renal function. (A) At Week 20 of the HFD, urine samples were collected overnight (15 h) for three consecutive days, and levels of urine albumin were determined in pooled urines. Albumin levels were normalized with corresponding urine creatinine levels to obtain the urine albumin-to-creatinine ratio (UACR) values. (B) Urine cystatin C levels were normalized with corresponding creatinine (CR) levels. The data are means ± SD from 3 pooled urine samples. ^a^P < 0.05 compared with the NFD group. ^b^P < 0.05 compared with the HFD/STZ group.

At week 21, mouse urine was collected for 15 h and the levels of urine albumin, cystatin C, total protein, and creatinine were determined in order to monitor renal function. The urine albumin-to-creatinine ratio (UACR) was elevated by 2.37–fold in the HFD/STZ group compared to the NFD group, which implies that CKD developed in these mice (Fig 2A). All treatment groups showed complete prevention of UACR increase, indicating that sarpogrelate, rosuvastatin, and their combination are all effective for CKD protection. Similarly, when another renal dysfunction marker, cystatin C was measured, the magnitude of the urine cystatin C-to-creatinine ratio significantly increased in the HFD/STD group, and drug treatment groups showed significant decreases (Fig 2B). Whereas, the urine protein-to-creatinine ratio was not elevated in the HFD/STZ group compared to the NFD group (data not shown). These results indicate that the combination of the HFD and a single low dose of STZ resulted in an UACR increase, which is a prototypical marker of diabetic CKD. In this animal model, sarpogrelate and rosuvastatin significantly ameliorated renal dysfunction and improved UACR and urinary cystatin C excretion.

All animals were terminated at Week 22. In final samples, the mean blood cholesterol level was significantly elevated the HFD/STZ mice when compared to the NFD mice. However, total triglyceride and BUN levels did not show significant increases (Table 1). The final body weights, kidney weights, and blood cholesterol were not altered by drug treatments. The sarpogrelate treatment group showed a triglyceride increase compared with the HFD/STZ group, raising a concern about the negative effect of sarpogrelate on blood lipid levels. However, we observed that the HFD-induced fatty liver development was substantially diminished by sarpogrelate treatment (unpublished data). In addition, our additional recent data indicated that sarpogrelate administration in HFD mice (for 12 weeks) did not affect blood triglyceride levels (unpublished data). Although further examination is required, these results indicate that the unwanted effects of sarpogrelate on blood lipid regulation might be minimal.

**Table 1:** After the completion of animal experiment, body weight and kidney weight were determined, and blood levels of cholesterol, triglycerides, and BUN were measured. Data are means ± SD

			Treatments		
variables	NFD	HFD/STZ	sarpogrelate	rosuvastatin	Sarpogrelate+rosuvastatin
	(n = 4)	(n = 5)	(n = 8)	(n = 7)	(n = 7)
Body weight (g)	30.78 ± 1.29	47.42 ± 3.11^a^	44.70 ± 5.11	42.69 ± 3.55	43.54 ± 6.17
Kidney weight (g)	0.211 ± 0.01	0.209 ± 0.01	0.206 ± 0.03	0.202 ± 0.03	0.203 ± 0.04
Cholesterol (mg/dl)	100.00 ± 13.37	144.60 ± 10.50^a^	155.63 ± 29.90	145.00 ± 22.39	132.29 ± 14.30
Triglyceride (mg/dl)	60.75 ± 13.72	61.80 ± 15.24	88.50 ± 15.95^b^	58.86 ± 14.35	76.57 ± 9.98
BUN (mg/dl)	24.75 ± 5.91	21.00 ± 3.74	21.38 ± 2.45	19.71 ± 3.90	20.00 ± 6.98

^a^P < 0.05 compared with the NFD group.

^b^P < 0.05 compared with the HFD/STZ group.

### Sarpogrelate and rosuvastatin ameliorate renal structural changes

**Fig 3:**
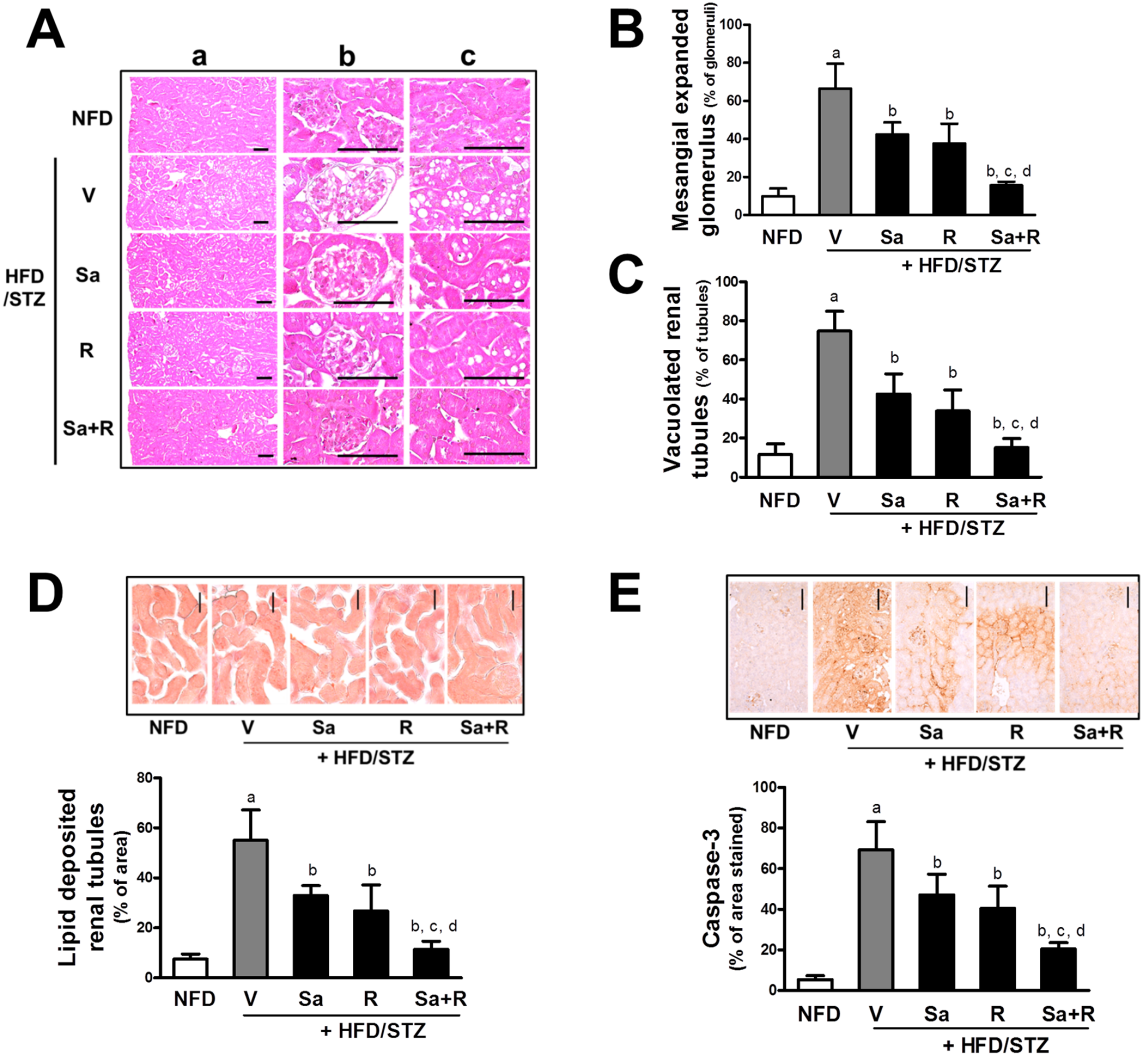
Effects of sarpogrelate and rosuvastatin on histomorphological changes in HFD/STZ mice. (A) H&E staining was performed in kidney tissues and representative histopathological images (cortex (a), glomerulus (b), and renal tubules (c)) are shown. Scale bar = 120 μm. (B) Numbers of glomeruli that display vasodilation and mesangial expansion were quantified with a computer-based automated image analyzer. (C) Numbers of tubules with vacuolation were quantified. (D) Oil red O staining was conducted and cortex areas with positive staining were quantified with a computer-based automated image analyzer. (E) Immunohistochemical staining was performed with caspase-3-specific antibody. Areas with caspase-3-positive staining were quantified. ^a^P < 0.05 compared with the NFD control group. ^b^P< 0.05 compared with the HFD/STZ group. ^c^P < 0.05 compared with the HFD/STZ + Sa group. ^d^P < 0.05 compared with the HFD/STZ + R group. Two histological fields for each kidney tissue were analyzed. All data represent means ± SD. Scale bar = 120 μm.

CKD is accompanied by several renal structural changes, including glomerular and mesangial expansion, glomerular endothelial proliferation, ECM accumulation, and tubular damage [42]. Histopathological examination of renal tissues showed glomerular structural changes: glomerular vasodilation and mesangial expansion were substantially elevated in the HFD/STZ group when compared to the NFD (Fig 3A and 3B). The HFD/STZ mice showed statistically significant increases in the numbers of vacuoles or lipid droplets deposition in renal tubules (Fig 3A, 3C and 3D). Consistent with these morphologic changes, the level of caspase 3-reactive regions, another marker of tubular damage, was elevated in HFD/STZ mice (Fig 3E). Notably, these histomorphometric and immunohistochemical changes were significantly attenuated by treatment with sarpogrelate or rosuvastatin. In particular, the combination of sarpogrelate and rosuvastatin displayed additive effects in all three of these parameters. These results indicate that the HFD/STZ-induced nephropathy was effectively ameliorated by sarpogrelate and rosuvastatin.

### Sarpogrelate and rosuvastatin suppress glomerular endothelial proliferation

**Fig 4:**
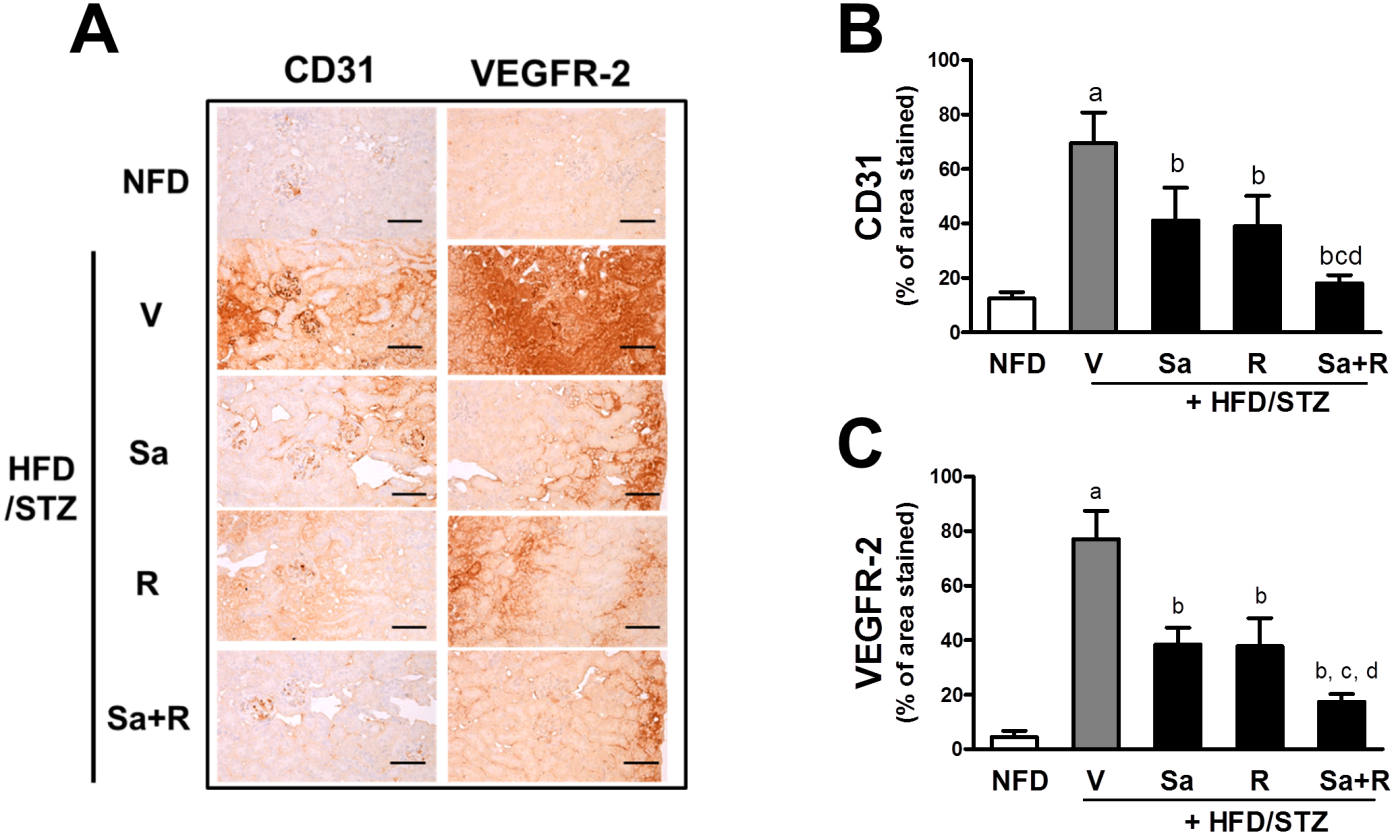
Amelioration effects of sarpogrelate and rosuvastatin on the expression of angiogenic markers CD31 and VEGFR-2. (A) Immunohistochemcial analyses were carried out with CD31- or VEGFR-2-specific antibodies and representative immunoreactive cortex regions are shown. (B-C) The CD31 (B) or VEGFR-2 (C)-positive cortex areas were quantified. ^a^P < 0.05 compared with the NFD control group. ^b^P< 0.05 compared with the HFD/STZ group. ^c^P < 0.05 compared with the HFD/STZ + Sa group. ^d^P < 0.05 compared with the HFD/STZ + R group. Two histological fields for each kidney tissue were analyzed. All data represent means ± SD. Scale bar = 120 μm.

Abnormal angiogenesis plays a pathological role in CKD [43, 44]. In order to monitor renal angiogenesis, the levels of CD31, a marker of endothelial cells, and VEGFR-2 were determined using immunohistochemical analysis. The HFD/STZ group showed increases in CD31 and VEGFR-2 protein levels, indicating that angiogenesis is aberrantly activated in the kidneys (Fig 4A–4C). On the other hand, the treatment of mice with sarpogrelate and rosuvastatin effectively attenuated CD31 and VEGFR-2 increases. Furthermore, the enhanced protective effects of sarpogrelate and rosuvastatin in combination were statistically significant.

### HFD/STZ-induced renal fibrosis is diminished by sarpogrelate and rosuvastatin

**Fig 5:**
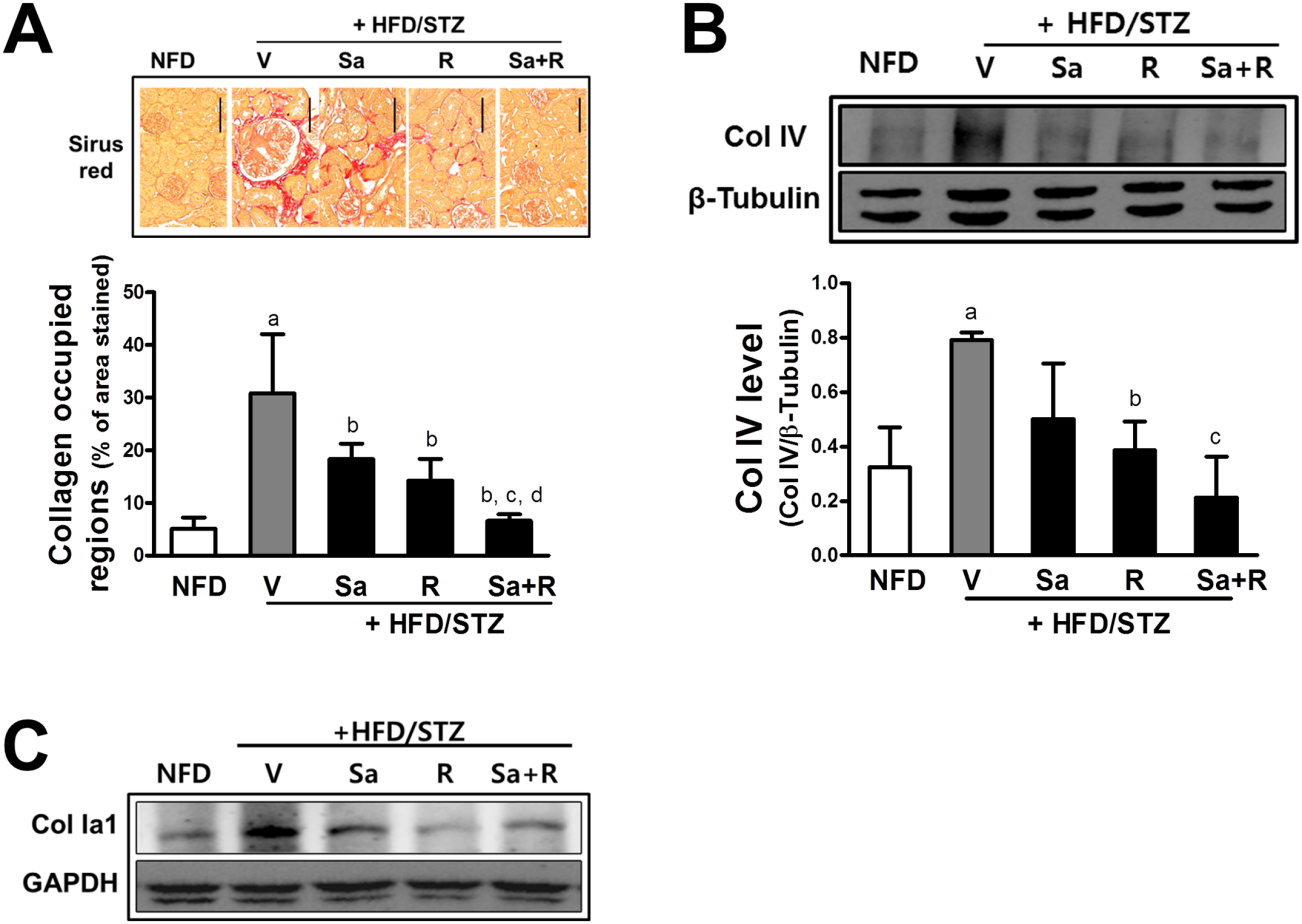
Attenuation of collagen deposition by sarpogrelate and rosuvastatin. (A) Kidney tissues were stained with Sirius red and cortex areas with collagen deposition were quantified. Two histological fields for each kidney tissue were analyzed. All data represent means ± SD. Scale bar = 120 μm (B) Protein levels of collagen type IV in kidney homogenates were assessed with western blot analysis. Data are means ± SD from three determinants of pooled samples. ^a^P < 0.05 compared with the NFD control group. ^b^P < 0.05 compared with the HFD/STZ group. ^c^P < 0.05 compared with the HFD/STZ +Sa group. ^d^P < 0.05 compared with the HFD/STZ + R group. (C) Protein levels of renal collagen type Ia1 were monitored with western blot analysis. Similar blots were obtained from three independent blots.

The deposition of ECM in the glomerulus and tubule, and progressive tublointerstitial fibrosis are major determinants of CKD [20]. In the HFD/STZ animals, cortex collagen deposition indicated by Sirus red staining was highly elevated compared to the NFD group (Fig 5A). In contrast, sarpogrelate and/or rosuvastatin reduced collagen staining. The HFD/STZ-induced increase in the collagen type IV and collagen type I levels were consistently reduced by sarpogrelate and rosuvastatin (Fig 5B and 5C).

**Fig 6:**
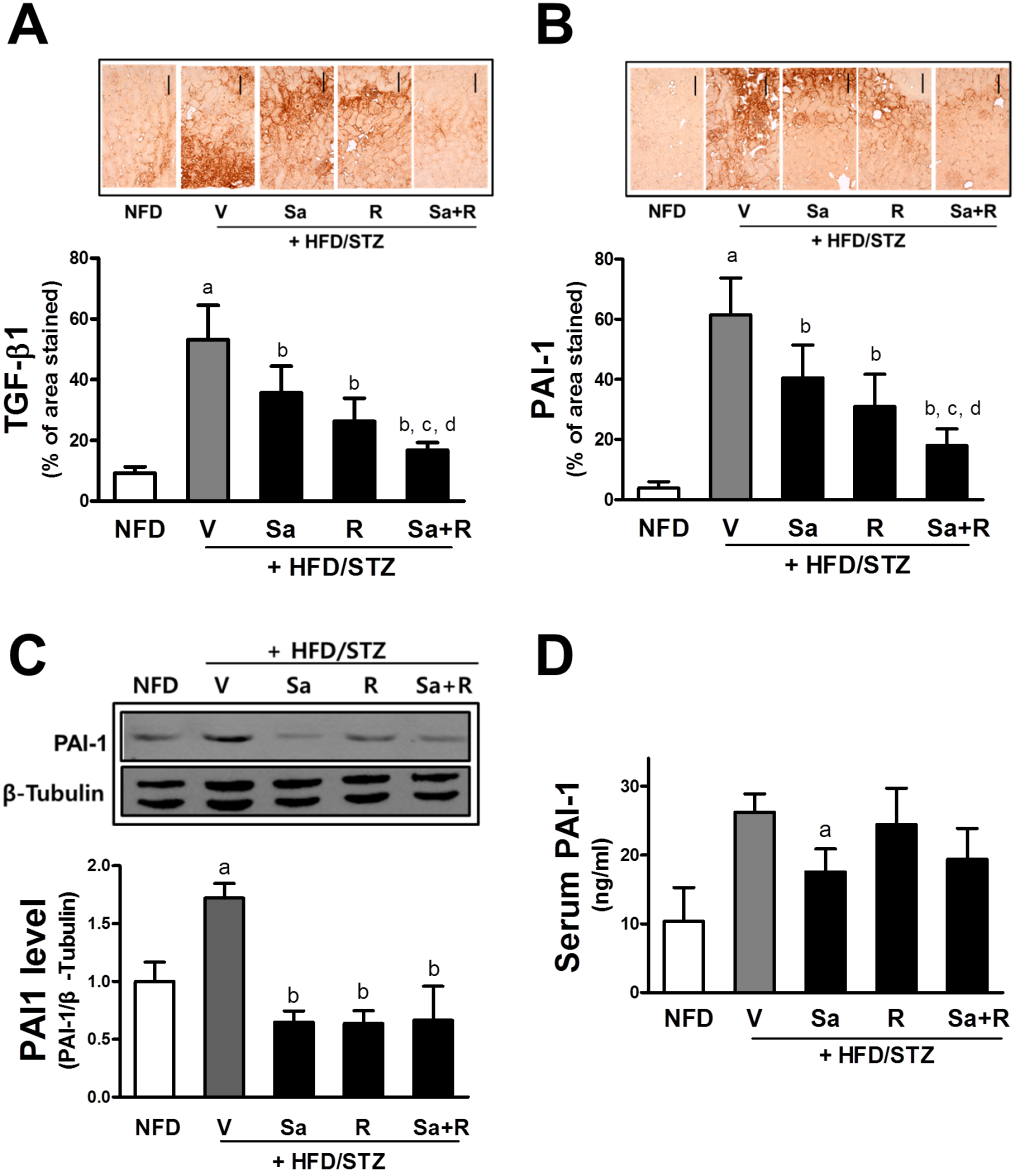
Inhibitory effects of sarpogrelate and rosuvastatin on pro-fibrotic factor TGF-β1 and PAI-1. (A-B) Immunohistological analysis was performed with TGF-β1 and PAI-1 antibodies. Cortex areas with TGF-β1 (A) and PAI-1 (B) expression were quantified. Two histological fields for each kidney tissue were analyzed. All data represent means ± SD. Scale bar = 120 μm. (C) Protein levels of PAI-1 in the kidney were determined with western blot analysis. Data are means ± SD from 3 measurement of pooled samples. ^a^P < 0.05 compared with NFD control group. ^b^P < 0.05 compared with the HFD/STZ group. ^c^P < 0.05 compared with the HFD/STZ + Sa group. ^d^P < 0.05 compared with the HFD/STZ + R group. (D) Serum levels of PAI-1 were monitored in blood samples that were obtained at the terminal step using an ELISA-based kit. Data are means ± SD from 4–8 samples. ^a^P < 0.05 compared with the NFD control group.

Accumulation of ECM is associated with enhanced levels of TGF-β1 and PAI-1 in the kidney [22]. Immunohistochemical analyses showed that HFD/STZ treatment elevates kidney levels of TGF-β1 and PAI-1 and that these increases were ameliorated by drug treatments (Fig 6A and 6B). Similarly to histomorphological improvements, the combination treatment with sarpogrelate and rosuvastatin showed significantly enhanced suppressions in TGF-β1 and PAI-1. Western blot analysis confirmed the suppressive effects of sarpogrelate rosuvastatin on PAI-1 increase (Fig 6C). At the same time, it was notable that serum level of PAI-1 was elevated 2.54-fold in mice with HFD/STZ treatment when compared with the NFD mice. This increase could be diminished by sarpogrelate treatment (Fig 6D), indicating that sarpogrelate modulates the circulating level of PAI-1 in HFD/STZ mice.

### Sarpogrelate inhibits PAI-1 expression in glomerular mesangial cells

**Fig 7:**
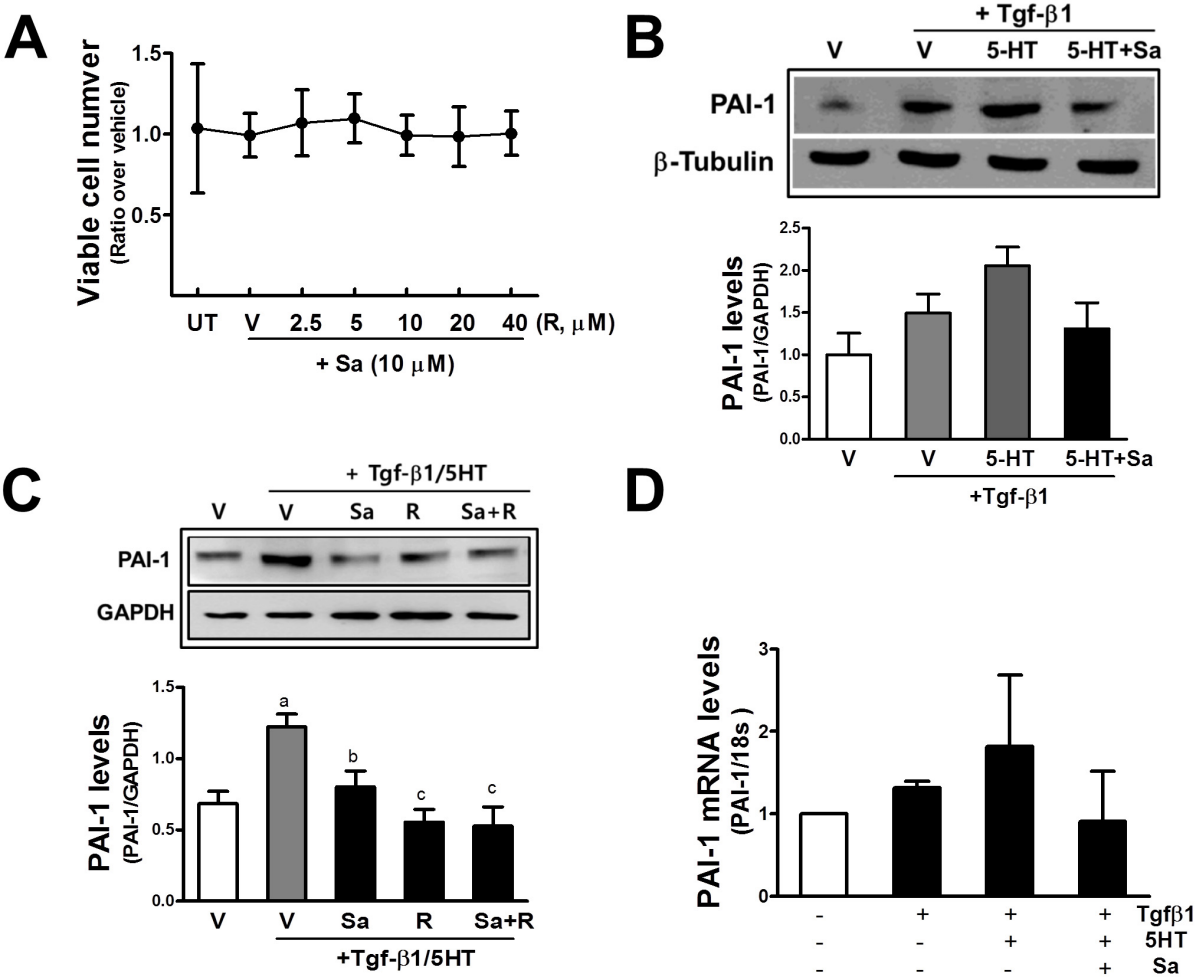
Suppressive effects of sarpogrelate on TGF-β1/5-HT-mediated PAI-1 increase in mesangial cells. (A) MES13 cells were incubated with sarpogrelate (Sa, 10 μM) along with vehicle (V, medium) or rosuvastatin (R, 2.5–40 μM) for 24 h, and viable cell numbers were assessed with MTT analysis. Data represent ratios with respect to untreated (UT) control group. Data are means ± SD from three experiments. (B) MES13 cells were pre-incubated with sarpogrelate (10 μM) for 6 h and then TGF-β1 (10 ng/ml) was additionally incubated for 24 h along with either vehicle (V) or 5-HT (10 μM). Levels of PAI-1 were assessed with western blotting. Data are means ± SD from three independent experiments. (C) MES13 cells were pretreated with sarpogrelate (10 μM), rosuvastatin (10 μM) or both for 6 h, and then TGF-β1 (10 ng/ml) and 5-HT (10 μM) were co-incubated for a further 24 h. Data are means ± SD from three independent experiments. (D) After the treatment of MES13 with sarpogrelate and TGF-β1/5-HT, transcript levels for PAI-1 were quantified with relative real-time RT-PCR analysis. Sarpogrelate was pre-incubated for 6 h and the TGF-β1/5-HT incubation was followed for a further 24 h. Data are means ± SD from three experiments.

Glomerular mesangial cells play a critical role in the maintenance of structure of the glomerular microvascular beds [45]. When these cells are confronted with detrimental stimuli such as metabolic and hemodynamic injuries, mesangial cells are activated to undergo proliferation and excessive ECM production [45]. Histomorphological analysis showed that sarpogrelate ameliorated mesangial expansion and glomerular enlargement, and reduced collagen depositions in the HFD/STZ mouse kidney. In order to have *in vitro* evidence, the murine mesangial cell line MES13 was incubated with sarpogrelate and rosuvastatin, and the levels of the pro-fibrotic factor PAI-1 were monitored. The incubation of MES13 with sarpogrelate (10 μM) and varied concentrations of rosuvastatin (2.5–40 μM) did not alter cell numbers, indicating no cytotoxic and proliferative effects (Fig 7A). The incubation of MES13 with TGF-β1 (10 ng/ml) for 24 h elevated PAI-1 level and this increase was further enhanced by the co-incubation with 5-HT (10 μM). Whereas, the TGF-β1/5-HT-induced PAI-1 increase was prevented by sarpogrelate pre-incubation (Fig 7B). Similarly, the level of PAI-1 that was elevated by co-incubation with TGF-β1 and 5-HT was blocked by sarpogrelate and rosuvastatin (10 μM) pre-incubation (Fig 7C). There was a trend for the TGF-β1/5-HT-inducible PAI-1 mRNA level to be diminished by sarpogrelate treatment; however, no statistical significance was obtained, implying the involvement of post-translational regulation mechanism (Fig 7D). Our results indicate that sarpogrelate reduced the PAI-1 protein level in mesangial cells and that this could be one of the molecular mechanisms mediating the renoprotective effect of sarpogrelate.

## Discussion

Although causative diseases are distinct, in all CKDs, a loss of normal nephrons results in compensatory glomerular hyperfiltration in the remaining nephrons. Elevated glomerular pressure causes endothelial injury and glomerular sclerosis, which ultimately lead to nephron malfunction. Glomerular injury and hyperfiltration give rise to albuminuria and the increase in urinary protein is associated with the initiation of inflammation and interstitial fibrosis [46]. Metabolic disorders, including obesity, diabetes, hyperlipidemia, and hypertension, are independent factors that are strongly associated with CKD incidence [1]. In addition, multimorbidity of these diseases increases the risk of CKD [12, 13]. Here, we investigated the potential beneficial effects of sarpogrelate and rosuvastatin on CKD in mice that were treated with 22-week HFD and a single and low dose of STZ as a model of metabolic disorder-induced CKD. In our animal model, renal morphological changes that are comparable to CKD were shown. These changes were mesangial expansion, glomerular endothelial proliferation, tubular damage, lipid accumulation, and collagen deposition. Consistent with these structural changes, urinary excretions of albumin and cystatin C were significantly enhanced, implying a reduction in renal function. Because cystatin C undergoes glomerular filtration, proximal tubular reabsorption, and eventually, complete catabolism in proximal tubular cells [47], and therefore, urinary cystatin C level can reflect tubular injury [48, 49]. Overall, the increased levels of UACR and the urinary cystatin C/creatinine ratio indicate dysfunctions in the glomerulus and proximal tubule, and confirm the establishment of the metabolic disorder-induced CKD model. Using this model, we demonstrated that the sarpogrelate and rosuvastatin treatments that were started after 8 weeks of HFD and STZ, effectively ameliorates CKD progression by improving renal morphological changes and preserving renal function. Notably, combination of sarpogrelate and rosuvastatin showed an additive renoprotective benefit in normalizing histological and immunopathological changes of CKD when compared to each treatment alone.

Sarpogrelate has been efficacious in various animal models of thrombosis, peripheral vascular disease, coronary artery diseases, and atherosclerosis [50]. Additionally, the potential beneficial effect of sarpogrelate on CKD has been suggested in several animal studies. Sarpogrelate administration for 8 weeks reduced albuminuria, glomerular platelet aggregation, and glomerular NO depletion in STZ-treated rats [51]. In mouse model of fibrosis in which mice were fed a 0.2% adenine diet for 6 weeks, sarpogrelate treatment (for 4 weeks) diminished tubulointerstitial fibrosis [52]. This study also demonstrated that renal fibrin deposition and PAI-1 increase were modulated by sarpogrelate treatment. Consistent with the animal evidence, sarpogrelate treatment improved albuminuria in patients with Type 2 diabetes especially, in the early-stage CKD [53]. Similarly, albuminuria was diminished by sarpogrelate in patients with diabetic CKD and atherosclerosis who were treated with an angiotensin II antagonist [54]. The renoprotective effects of sarpogrelate can be attributed to the blockade of the deleterious effects of the 5-HT_2A_ receptor, including vasoconstriction, vascular proliferation, and platelet aggregation [29]. In particular, the involvement of 5-HT in nephropathy has been suggested in several reports. Blood 5-HT levels were high in diabetic patients, indicating that 5-HT-mediated platelet aggregation was increased in these patients [31, 32]. Additionally, the sensitivity to 5-HT-induced vasoconstriction is significantly elevated in diabetic patients, implying the role of 5-HT in glomerular endothelial injury [55]. Moreover, 5-HT was shown to increase collagen IV production in human mesangial cells through protein kinase C and TGF-β1 activation [56].

Advantageous effects of statins on the progression of CKD have been observed in clinical observations. The Prospective Evaluation of Proteinuria and Renal Function in Diabetic Patients with Progressive Renal Disease (PLANET) studies showed that atorvastatin for 52 days lowered proteinuria in patients with hypercholesterolemia and diabetes [57]. A collective analysis of statin studies that had endpoints of CKD and cardiovascular diseases led to a conclusion that statin treatments improved microalbuminuria, proteinuria, and GFR in early stages of CKD patients [58]. Based on the association of high lipid levels with CKD prevalence, these beneficial effects on CKD can be attributed to the management of dyslipidemia [35]. However, several animal experiments demonstrated that statins exerted renal benefits that is independent on the lipid-lowering effect. Rosuvastatin treatment for 8 weeks reduced the excretion of urinary albumin and suppressed glomerular hypertrophy in *db/db* mice even though blood lipid levels were not altered by drug treatment [59]. In *Apo-E* knockout mice, rosuvastatin administration for 20 weeks showed renoprotective effects that are irrelevant to the lipid-lowering effect [60]. This study showed that rosuvastatin diminished the expression of NADPH oxidase 4 (NOX4) and receptor for advanced glycation and products (RAGE) in the kidney. Similarly to these reports, although rosuvastatin was effective in ameliorating CKD progression, rosuvastatin treatment did not affect blood cholesterol levels in our experimental design. This implies that additional pleiotropic actions might contribute renal benefits of rosuvastatin. Indeed, rosuvastatin treatment elevated the expression of heme oxygenase-1 (Ho-1), a renoprotective anti-inflammatory protein, in our kidney samples (unpublished data). In addition, rosuvastatin treatment suppressed pro-fibrotic PAI-1 levels in the kidney as well as mesangial cells.

PAI-1 blocks plasmin-induced fibrinolysis by inhibiting tissue plasminogen activator (tPA) and urokinase plasminogen activator (uPA). In addition to this major role in fibrinolysis, PAI-1 is involved in ECM homeostasis by inhibiting the plasmin-induced ECM degradation [23, 24]. The direct link between PAI-1 and renal fibrosis can be drawn from studies with *pai-1* null mice. Unlike wild-type animals, collagen accumulation and interstitial fibrosis were significantly reduced in *pai-1* null animals following unilateral ureteral obstruction [61]. PAI-1-mediated renal fibrosis is strongly associated with TGF-β1; PAI-1 level and interstitial collagen deposition were highly increased in *TGF-β1* transgenic mice and on the contrary, *pai-1* deficiency prevented TGF-β1-induced renal fibrosis and ECM accumulation [62]. Our results indicate that anti-fibrotic effect of sarpogrelate and rosuvastatin can be attributed to the inhibitory effects on PAI-1 and TGF-β1. Sarpogrelate and rosuvastatin treatment reduced collagen deposition and suppressed HFD/STZ-induced PAI-1 and TGF-β1 increases in the kidney. In addition, as shown *in vitro*, sarpogrelate and rosuvastatin incubation in murine mesangial cells suppressed TGF-β1/5-HT-induced PAI-1 elevation. Interestingly, in addition to tissue levels, sarpogrelate treatment diminished blood PAI-1 levels that were substantially elevated in HFD/STZ animals. In humans, there are lines of evidence indicating that increased circulating PAI-1 level is related with atherothrombosis incidence [28]. Moreover, blood PAI-1 levels were higher in obese patients with metabolic syndrome and in diabetic patients [26]. Considering that subjects with metabolic diseases show a higher incidence of cardiovascular diseases, increased circulating PAI-1 levels might be a causative link between metabolic syndrome and atherothrombotic diseases, and therefore, the inhibitory effect of sarpogrelate on circulating PAI-1 may imply additional benefits of sarpogrelate for the protection of CKD-induced cardiovascular complications.

In conclusion, we demonstrated that sarpogrelate and rosuvastatin could effectively ameliorate HFD/STZ-induced CKD progression by improving histopathological changes in the glomerulus and tubules, and attenuating interstitial fibrosis, albuminuria, and urinary cystatin C excretion. These drugs inhibited pro-fibrotic PAI-1 expression in HFD/STZ mouse kidney and mesangial cells, indicating a mechanistic basis for the observed histopathological effects. Moreover, combinatorial treatment with sarpogrelate and rosuvastatin was relatively more effective in ameliorating histopathological changes when compared to the single drug treatment; therefore, sarpogrelate may have potential benefits for the control of CKD in patients with metabolic disorders, particularly in conjunction with statins therapy.
